# Implementing therapist‐guided internet‐delivered cognitive behaviour therapy for obsessive–compulsive disorder in the UK’s IAPT programme: A pilot trial

**DOI:** 10.1111/bjc.12365

**Published:** 2022-03-22

**Authors:** Oskar Flygare, Lina Lundström, Erik Andersson, David Mataix‐Cols, Christian Rück

**Affiliations:** ^1^ 27106 Department of Clinical Neuroscience Centre for Psychiatry Research Karolinska Institutet Stockholm Sweden; ^2^ Stockholm Health Care Services Stockholm Sweden; ^3^ CAP Research Centre Stockholm Health Care Services Stockholm Sweden

**Keywords:** internet‐delivered cognitive behaviour therapy, obsessive‐compuslive disorder, Improving Access to Psychological Therapies, OCD‐NET

## Abstract

**Objectives:**

Digital therapies such as internet‐delivered cognitive behaviour therapy (ICBT) can improve treatment access for patients with common mental disorders, but are rarely used in the Improving Access to Psychological Therapies (IAPT) programme in the United Kingdom. The objective of this study was to evaluate an evidence‐based ICBT intervention for obsessive‐compulsive disorder (OCD‐NET) in three IAPT services in an open trial.

**Methods:**

Consecutively referred patients with a primary diagnosis of OCD (*n *= 474) were offered OCD‐NET. Symptoms of OCD, depression, anxiety, and level of functioning were measured weekly throughout treatment.

**Results:**

In the full intention to treat sample (*n *= 474), the intervention was associated with large reductions in self‐reported OCD symptoms (*d* = 1.77), anxiety (*d* = 1.55) and depression (*d* = 0.8), as well as improvements in functional impairment (*d* = 0.51 to 0.72). Further, 35% of participants were in recovery at their last assessment, 25% achieved reliable improvement and 15% met criteria for both recovery and improvement. Among participants completing at least 4 modules (*n *= 261), corresponding to an adequate ‘dose’ of treatment, the rates of recovery (44%), reliable improvement (34%) and reliable recovery (21%) were higher. A majority of participants were satisfied with the online treatment and found the online materials helpful.

**Conclusions:**

OCD‐NET is an effective treatment when delivered in regular care within the IAPT system. Challenges associated with implementing ICBT in regular health care are discussed.

**Practitioner Points**

- Internet‐delivered cognitive behaviour therapy is an effective treatment option for individuals with obsessive–compulsive disorder.
- Health care providers should ensure that therapists receive adequate training and support to deliver internet‐based treatment effectively.

## INTRODUCTION

Obsessive–compulsive disorder (OCD) is characterized by intrusive and unwanted thoughts (obsessions), and repetitive behaviours or mental acts aimed at neutralizing the anxiety caused by intrusive thoughts (compulsions) (American Psychiatric Association, 2013). OCD has a lifetime prevalence of 1.3% and typically onsets before the age of 25 years (Fawcett et al., 2020; Fullana et al., 2009). It is often chronic if left untreated (Mataix‐Cols et al., 2002; Skoog & Skoog, 1999) and is associated with pervasive educational impairment (Pérez‐Vigil et al., 2018), labour market marginalization (Pérez‐Vigil et al., 2018), and low quality of life (Huppert et al., 2009).

Cognitive behaviour therapy (CBT) is a recommended first‐line treatment for OCD (OCD, 2005; Öst et al., 2015), but only a minority of patients receive this treatment (Goodwin et al., 2002; Marques et al., 2010). Common barriers to treatment include logistic and financial barriers (e.g., problems with transportation or scheduling), stigma and shame (e.g., being afraid of what others would think), lack of access to adequately trained therapists, and concerns about the cost of treatment (Goodwin et al., 2002; Marques et al., 2010). Digital therapies such as internet‐delivered CBT (ICBT) are able to overcome several barriers to treatment as they can be delivered remotely and at flexible times. For therapists and health care services, the structured format of ICBT means that less therapist time is required per patient, making it a cost‐effective alternative or complement to conventional in‐person therapies (Andersson et al., 2015; Hedman et al., 2012). In the last 10 years, multiple ICBT programmes for OCD have been developed (Schröder et al., 2020; Vogel et al., 2014; Wootton et al., 2013) and evaluated in clinical practice (Lovell et al., 2017; Luu et al., 2020; Titov et al., 2017; Wootton et al., 2021). A recent meta‐analysis concluded that ICBT is an efficacious and promising treatment option for individuals with OCD (Hoppen et al., 2021). Among these, a Swedish internet‐delivered CBT programme called OCD‐NET has been extensively evaluated in multiple open (Andersson et al., 2011; Patel et al., 2018) and randomized controlled trials (Andersson et al., 2012, 2015), with sustained gains up to 2 years after treatment (Andersson et al., 2014).

The Improving Access to Psychological Therapies (IAPT) programme aims to increase access to evidence‐based treatment for depression and anxiety disorders in the United Kingdom by training new therapists and deploying them in services focused on mental health (Clark, 2011, 2018). Since its inception in 2008, IAPT has expanded to treat over 600,000 patients yearly with evidence‐based psychological treatments, of which 19,000 received treatment for OCD in 2019–2020 (Team I & Digital N, 2020). An important feature of IAPT is that therapy outcomes are scored using validated questionnaires, with 98% of patients having pre‐treatment and post‐assessment data. IAPT also provides publicly available outcome data (Clark et al., 2018). A survey of 191 IAPT services found that only 24% were using digital treatments recommended by the National Institute for Health and Care Excellence (NICE) (Bennion et al., 2017). Further, among the 600,000 treatments completed in 2019–2020, only 7,600 (or 1.3%) were therapist‐guided ICBT treatments (Team I & Digital N, 2020). Initiatives are now underway to evaluate several ICBT treatments within IAPT, and potentially improve treatment access further (Richards et al., 2018). Key considerations for evaluations of ICBT in IAPT are (1) choosing the right interventions (i.e., those that have a solid evidence‐base and are easy to use), (2) ensuring adequate therapist training, and (3) practical and organizational factors related to the implementation of the digital treatments (Thew, 2020).

This study piloted the implementation of OCD‐NET at three IAPT services during 2019–2021. Prior to the evaluation in practice, OCD‐NET underwent a rigorous evaluation by NICE where the scientific evidence, intended place in therapy, key uncertainties, as well as costs and resource impact were evaluated (NICE, 2019). OCD‐NET was found to be suitable for evaluation in practice within the IAPT system, the key uncertainty in evidence being that there were no direct comparisons with face‐to‐face CBT. OCD‐NET was appraised to lead to cost savings compared to individual CBT (NICE, 2019). This study describes the effectiveness and acceptability of OCD‐NET when implemented at three IAPT services, and discusses practical and organizational factors from the evaluation.

## METHODS

### Study design

This study was an open trial of the effectiveness of OCD‐NET when delivered at three IAPT services: Steps2Wellbeing Dorset, Let's Talk Gloucestershire, and Let's Talk Haringey in London. Participants obtained information about the study, what was involved in participating, the treatment, and how to proceed with participation in telephone screenings at the three services. Participants also provided written informed consent for participating in the evaluation when accessing the OCD‐NET platform for the first time.

### Procedure

After evaluation of the evidence for OCD‐NET and development of the therapy platform to meet usability and security standards by NICE and IAPT, a 2‐year evaluation in practice took place between September 2019 and September 2021 (NICE, 2019). In August 2019, 1‐day training sessions were held by authors OF and CR at the three services selected to participate. The training included information about OCD‐NET and the techniques used, mainly exposure with response prevention. Further, therapists and clinical leads were given access to therapist accounts and test patient accounts to practice the day‐to‐day use of the treatment platform. These were presented as clinical scenarios which included writing and responding to messages, reviewing homework and worksheets, managing suicidal ideation notifications, and methods for supervision on the platform. A comprehensive online therapist manual was also developed where therapists could learn more about using the platform and best practices in ICBT. Additional training sessions were held remotely via videoconferencing with the project leader (OF) as needed, for example, due to therapist turnover or lack of previous experience in treating OCD among newly trained therapists. Throughout the evaluation, OCD‐NET therapists were supervised via the platform and bi‐weekly supervision sessions were held via videoconferencing where therapists could discuss issues related to using the platform as well as questions or difficulties in the ongoing treatments. As therapists gained more experience using the treatment platform and providing effective support to patients, videoconference supervision was stepped down to occur once per month.

### Participants

Consecutively referred patients at the three sites with a primary diagnosis of OCD were offered OCD‐NET as a treatment option after undergoing a standardized IAPT assessment. The assessment includes information about the IAPT service, exploration of the patient's presenting problem(s), identification of the appropriate problem descriptors using ICD‐10 codes, a standardized risk assessment, and completion of the IAPT Data Set including diagnosis‐specific outcome measures. The assessments were conducted by Psychological Well‐being Practitioners (PWPs) or High‐Intensity CBT therapists (The National Collaborating Centre for Mental Health, 2021). There were no restrictions regarding psychotropic medication, the severity of OCD symptoms, or comorbidity with depression or anxiety disorders as long as OCD was the primary diagnosis. Exclusion criteria were moderate to high suicidal ideation that would require frequent follow‐up (i.e., clear plans or intent to act on suicidal thoughts), low motivation to participate (e.g., does not believe the therapy will be helpful at all) or attention deficits that would interfere with reading the treatment texts (e.g, unmedicated attention deficit hyperactivity disorder, self‐reported difficulties reading books or newspapers), insufficient time to work on the treatment (about 45 minutes per day in the most intense phase), severe depression (i.e., PHQ‐9 total score 20–27 with marked loss of daily functioning), another ongoing psychological treatment or prior treatment for OCD at the service, or a severe mental disorder that would interfere with treatment (e.g., unmedicated psychosis or bipolar disorder and untreated substance use disorder).

### Therapist eligibility

All interested therapists were eligible to participate, regardless of prior experience using digital therapies or treating OCD. Prior to the OCD‐NET evaluation, the services had treated OCD at step 2 using PWPs at the Dorset service or step 3 using high‐intensity CBT therapists (HI‐CBT) at the Gloucestershire and Haringey services, and the therapists involved in the evaluation were thus a mix of PWPs and HI‐CBT therapists. To facilitate the development of skills and efficient use of the platform, it was recommended that each therapist had a case‐load of 4–8 concurrent patients in OCD‐NET. The therapists had been working within the IAPT system between 1 and 12 years (mean = 4 years). Previous experience in treating OCD varied by level of training with 71% of PWPs in Dorset having treated no or fewer than five OCD patients, while all HI‐CBT therapists in Gloucestershire and Haringey had treated OCD and 38% of therapists had seen more than ten patients prior to this study.

### Intervention

OCD‐NET is a 10‐week therapist‐guided, internet‐delivered intervention for OCD. There are 10 treatment modules that include texts and images (e.g., what is OCD, introduction to exposure with response prevention), along with homework assignments and worksheets. As with validated treatment protocols of face‐to‐face CBT for OCD, the emphasis is on exposure with response prevention (Foa et al., 2012), and the difference is in the mode of delivery rather than content. To access OCD‐NET, patients log on to a secure website where they can read module texts, respond to questionnaires and homework exercises, fill out worksheets (e.g., evaluating an exposure task), as well as write and respond to messages from the therapist. Therapists provide daily support by writing and responding to messages, reviewing homework assignments and worksheet entries, and prompting the patients to engage with exposure tasks. For more information regarding the treatment, please see descriptions in previous randomized controlled trials of OCD‐NET (Andersson et al., 2012, 2015; Rück et al., 2018).

### Primary outcome measure

The primary outcome measure was the Obsessive–Compulsive Inventory‐Revised (OCI‐R), a self‐rated questionnaire consisting of 18 items assessing various domains of OCD symptoms (Foa et al., 2002). Each item is scored 0–4 on a 5‐point Likert scale, yielding a total score of 0–72. The OCI‐R has adequate internal validity (Cronbach's α = .81), a 2‐week test–retest reliability of 0.82, as well as a moderate convergent validity with the clinician‐rated Yale‐Brown Obsessive–Compulsive Scale (Goodman et al., 1989) of *r* = .53 (Foa et al., 2002). In the current sample, the internal validity was good (α = .87 [95% CI 0.86 to 0.89]). The OCI‐R is sensitive to changes in OCD symptoms during treatment (Abramowitz et al., 2005) and it can distinguish between patients diagnosed with OCD from those with other anxiety disorders (AUC = 0.81) (Abramowitz & Deacon, 2006). A cut‐off of 17 was used as the threshold for recovery in this trial, based on previous analyses of IAPT data (Veale et al., 2016) and the threshold for a reliable change was 13 points (Veale et al., 2016). The OCI‐R was administered via the treatment platform pre‐treatment, weekly during treatment, as well as post‐treatment.

### Secondary outcome measures

The Patient Health Questionnaire‐9 (PHQ‐9), Generalized Anxiety Disorder 7‐item scale (GAD‐7), and Work and Social Adjustment Scale (WSAS) are included in the IAPT outcome monitoring system. National averages in pre‐treatment and post‐treatment values as well as effect sizes are available as benchmarks (England PH, 2021). A modified version of the Patient Experience Questionnaire (PEQ) that includes additional questions regarding digital therapy was included post‐treatment.

#### Patient health questionnaire‐9

The PHQ‐9 is a self‐rated questionnaire for major depressive disorder (Kroenke et al., 2001). Nine items (e.g., little interest in doing things) are scored on a scale from 0 ‘not at all’ to 3 ‘nearly every day’ during the last 2 weeks, yielding a total score of 0–27 (Kroenke et al., 2001). The PHQ‐9 has excellent internal validity (Cronbach's α = .89) (Kroenke et al., 2001), which was also seen in the current sample (α = .86 [95% CI 0.84 to 0.88]). A cut‐off of ≥10 points distinguishes patients with major depressive disorder with a sensitivity of 88% and a specificity of 88% and was used as the cut‐off for recovery in this trial (Kroenke et al., 2001). The threshold for reliable change was 6 points. In this trial, the PHQ‐9 was administered at pre‐treatment, weekly throughout treatment, and post‐treatment.

#### Generalized anxiety disorder 7

The GAD‐7 is a 7‐item anxiety scale that measures common symptoms associated with generalized anxiety disorder in the last 2 weeks (Spitzer et al., 2006). Each item ranges from 0 ‘not at all’ to 3 ‘nearly every day’, resulting in a total score of 0–21. The GAD‐7 has excellent internal consistency (Cronbach's α = .92) and convergent validity (correlation with the Beck Anxiety Inventory *r* = .72) (Spitzer et al., 2006). In the current sample, internal consistency on the GAD‐7 was excellent (α = .87 [95% CI 0.85 to 0.89]). A score of ≥8 was used as the cut‐off for caseness, and the reliable change threshold was 4 points. The GAD‐7 was administered at pre‐treatment, weekly throughout treatment, and post‐treatment.

#### Work and social adjustment scale

The WSAS measures functional impairment in five domains: work, home management, social leisure activities, private leisure activities, and close relationships (Mundt et al., 2002). Each domain is scored from 0 ‘no impairment’ to 8 ‘very severe impairment’. Internal consistency ranges from Cronbach's α 0.70–0.94 for the separate items (α ranged from 0.76 to 0.82 in the current sample), and the 2‐week test–retest reliability is *r* = .73 (Mundt et al., 2002). Similar to the other self‐rated measures, the WSAS was administered weekly from pre‐treatment to post‐treatment.

#### Patient experience questionnaire

The modified version of the PEQ evaluates patients’ experiences with the IAPT service in general, with additional questions about the online therapy in particular. First, patients answer five questions regarding the service on a 5‐point Likert scale ranging from ‘At all Times’ to ‘Never’, for example, ‘Did you feel involved in making choices about your treatment and care?’ Next, patients rate their satisfaction with the online therapy on a 5‐point Likert scale, ranging from ‘Completely Satisfied’ to ‘Not at All Satisfied’. The four questions included are ‘How satisfied were you with your online therapy?’, ‘How helpful did you find the online self‐help materials?’, ‘How easy was the online therapy to use?’, and ‘How easy was it to communicate with your therapist?’. The PEQ was administered at post‐treatment.

#### Recovery, improvement, and deterioration

A common measure of treatment effects in the IAPT system is recovery rates, which is how many patients score above *caseness*, for example, scoring 17 or more on the OCI‐R, at pre‐treatment and have a score below the caseness threshold on their last appointment. The overall goal is that 50% of patients reach recovery after treatment (Clark, 2018). In this trial, a patient was considered to be in recovery if they had completed at least two weekly assessments and the last available total scores on the OCI‐R and PHQ‐9 were both below caseness cut‐offs (The National Collaborating Centre for Mental Health, 2021).

Reliable improvement is defined as a clinically significant reduction in symptoms between the first and last scores, and deterioration occurs when there is a clinically significant increase in symptoms. To meet IAPT criteria for reliable improvement or deterioration, at least one of OCI‐R or PHQ‐9 has to move into the range for improvement/deterioration while the other measure moves in the same direction or is unchanged. No reliable change occurs when one measurement meets criteria for reliable improvement and the other meets criteria for reliable deterioration, or when neither measure has a change that exceeds the reliable change threshold (The National Collaborating Centre for Mental Health, 2021).

Recovery and reliable improvement can be combined to create an overall measure of *reliable recovery*, that is, patients that meet criteria for both recovery and reliable improvement (The National Collaborating Centre for Mental Health, 2021).

#### Engagement and satisfaction

Participant engagement was measured by counting the number of messages sent and received by participants, as well as module completion (i.e., whether participants read the main text of a certain module during treatment). Participants who reached module 4 were classified as treatment completers since they had reached the core intervention exposure with response prevention.

A modified version of the PEQ was included in the post‐treatment assessment, with added questions about the digital therapy (The National Collaborating Centre for Mental Health, 2021). Participants were asked to rate satisfaction with the online therapy, whether the online materials were helpful, to what degree the online therapy was easy to use, and the ease of communicating with their therapist using the online therapy. Each question was rated on a 5‐point Likert scale ranging from ‘Completely Satisfied’ to ‘Not at All Satisfied’.

### Risk management

Therapists monitored weekly responses to questionnaires throughout treatment and discussed any unexpected changes with patients (e.g., a sudden increase in OCD symptoms). Further, if a participant indicated heightened suicidal ideation on the PHQ‐9 question 9 (‘Thoughts that you would be better off dead or of hurting yourself in some way’) by responding ‘More than half the days’ or ‘Nearly every day’, an automatic monitoring system on the treatment platform flagged the patient and therapists conducted a structured suicide risk assessment via telephone. After the structured suicide risk assessment, the participant received additional calls as needed to monitor risk and was otherwise managed according to local routines in place at each IAPT service.

### Statistical analyses

The primary and secondary outcomes were fitted using mixed‐effects linear regression models with a fixed effect of time and a random intercept for each participant. These models included all participants according to intention to treat, and missing data was estimated using maximum likelihood. Standardized effect sizes were calculated based on the least‐squares means using the residual standard deviation of the random effects as sigma. Recovery, reliable improvement, and reliable recovery were calculated based on the first and last assessments for participants who had responded to questionnaires on two or more occasions. Post‐hoc tests for site differences on baseline characteristics were conducted using analysis of variance for continuous variables and chi‐square test of independence for categorical variables. Sensitivity analyses were conducted on treatment completers (i.e., participants who reached module 4). Because the amount of previous experience treating OCD varied by level of training, post‐hoc analyses were done by including therapist training as a covariate in the main OCI‐R model, as well as comparing the OCI‐R improvement between the first five and subsequent participants for each therapist to evaluate the effect of becoming more experienced in using OCD‐NET. R version 4.0.5 was used for statistical analyses (R Core Team. R, 2021). The scripts used for statistical analyses are available on the Open Science Framework (https://osf.io/cakxb/).

## RESULTS

### Participant characteristics

The majority of participants were female and reported clinically significant symptoms of OCD for 8 years prior to participating in the study. The most common comorbid condition was major depressive disorder and 29% of participants were taking antidepressant medication (see Table 1). Post‐hoc analyses of site differences showed that participants at the Haringey site were more likely to not be in a relationship (*p* = .002), and had higher educational attainment (*p *< .001) compared to the other two sites. Having a diagnosis of post‐traumatic stress disorder was more common at the Gloucestershire site (6.5%) compared to Dorset (1%) and Haringey (2.8%) (*p* = .008) and participants at the Gloucestershire site were also more likely to be currently taking antidepressant medication with selective serotonin reuptake inhibitor (38.9%) compared to the Dorset (27.6%) and Haringey (19.4%) sites (*p* = .013). One participant ended treatment prematurely due to a sudden increase in suicidal ideation and was moved to face‐to‐face treatment with more frequent follow‐up.

**TABLE 1:** Participant characteristics

	*n* (%)
Age in years, mean (*SD*)	30.02 (10.62)
Sex, female	303 (73%)
Relationship status	
Single	194 (41%)
Partner or married	207 (44%)
Divorced	6 (1%)
Other or prefer not to answer	67 (14%)
Occupational status	
Working	290 (70%)
Student	61 (15%)
Retired	3 (1%)
Unemployed	59 (14%)
Disability pension	1
Currently on sick leave	26 (6%)
Highest attained education	
Primary school	4 (1%)
High school	146 (35%)
Bachelor's degree	223 (54%)
Master's degree	37 (9%)
Doctorate	4 (1%)
Years with OCD, mean (SD)	8.13 (8.55)
Previous CBT for OCD	107 (26%)
Comorbid disorders	
Major depressive disorder	106 (22%)
Generalized anxiety disorder	20 (4%)
Social anxiety disorder	4 (1%)
Panic disorder	14 (3%)
PTSD	12 (3%)
Autism spectrum disorder	12 (3%)
ADHD	5 (1%)
Anorexia	9 (2%)
Bulimia	5 (1%)
Other^a^	15 (3%)
Current medication	
SSRI	137 (29%)
Other^b^	13 (3%)

Abbreviations: ADHD, attention‐deficit hyperactivity disorder; CBT, cognitive behaviour therapy; OCD, obsessive–compulsive disorder; PTSD, post‐traumatic stress disorder; SSRI, selective serotonin reuptake inhibitor.

^a^ Other disorders include (*n*): Agoraphobia (1), Body dysmorphic disorder (2), Bipolar disorder (3), Borderline personality disorder (2), Emetophobia (2), Health anxiety disorder (2), Insomnia (1), Schizophrenia (1), Tic disorder (1).

^b^ Other medications include (*n*): Other antidepressants such as Selective Noradrenaline Reuptake Inhibitors (6), Beta‐adrenergic blocking agents (3), Bensodiazepines (2), Central stimulants (1), Anti‐psychotic medication (1).

### Module completion

**FIGURE 1:**
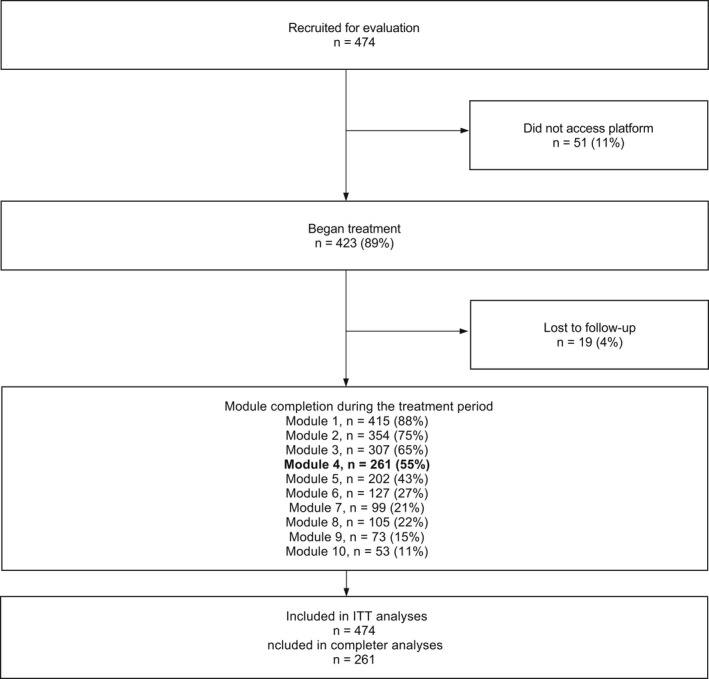
Participant flow through the study

A total of 261 (55%) participants were considered treatment completers as exposure with response prevention is first introduced in Module 4. The number of participants who accessed each module during the treatment period is shown in Figure 1.

### Primary outcome

In ITT analyses (*n* = 474), the OCI‐R decreased from 31.1 at pre‐treatment to 21.07 at post‐treatment (mean reduction −10.04 points, *d* = 1.77 [1.58 to 1.97]), a reduction that was statistically significant (*F* [11, 2644.45] = 77.49, *p* < .001). Sensitivity analyses were conducted on the 261 participants who reached module 4 within the treatment period and were considered completers as they had been introduced to exposure with response prevention. The estimated pre–post change was larger among treatment completers (mean reduction −11.1 points, *d* = 2.04 [1.83 to 2.26]).

**FIGURE 2:**
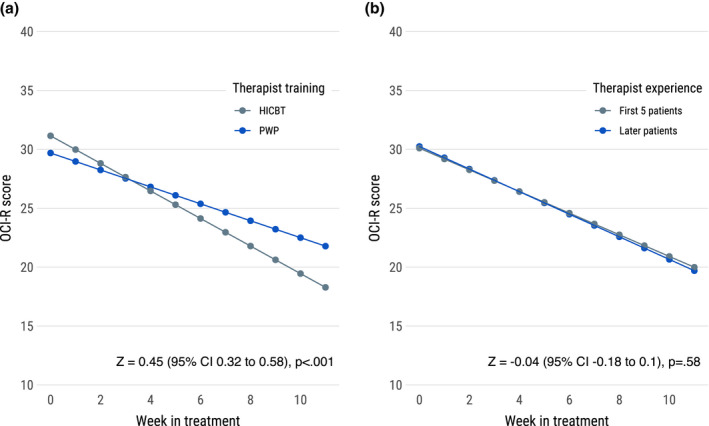
(a) OCI‐R reduction over time by level of therapist training. (b) OCI‐R reduction over time by level of therapist experience with OCD‐NET. Abbreviations: HICBT, High‐Intensity CBT therapist; OCI‐R, Obsessive–compulsive Inventory‐Revised; PWP, Psychological Well‐being Practitioner

Post‐hoc analyses showed that therapist training, but not increased experience in using OCD‐NET, moderated results. There was a statistically significant interaction effect between level of therapist training and weekly reduction of OCI‐R scores, indicating that participants receiving treatment from PWP therapists improved significantly less over time (Z = 0.45, 95% CI 0.32 to 0.58, *p* < .001) as seen in Figure 2a. In contrast, there was no statistically significant effect of experience with using OCD‐NET as outcomes were identical when comparing the first 5 to subsequent patients for each therapist (Z = −0.04, 95% CI −0.18 to 0.1, *p* = .58), see Figure 2b.

### Secondary outcomes

Symptoms of anxiety on the GAD‐7 also decreased during treatment, from 13.16 at pre‐treatment to 8.7 after treatment. The mean reduction was −4.46 points (*d* = 1.55 [1.36 to 1.73]) in the full sample and −4.93 points (*d* = 1.71 [1.5 to 1.92]) when only analysing participants classified as treatment completers. This reduction can be compared to publicly available data on OCD patients in all of IAPT, which reported a mean reduction from 15.1 to 8.8 (*d* = 1.5) (Team I & Digital N, 2020).

The average PHQ‐9 score at pre‐treatment was 11.47 and decreased to 8.32 at post‐treatment (mean reduction −3.15, *d* = 1.04 [0.85 to 1.23]). Among treatment completers, the mean pre–post reduction was −3.65 (*d* = 1.24 [1.03 to 1.45]). The corresponding reduction for all OCD patients in IAPT is from 13.5 at pre‐treatment to 8.3 at post‐treatment (*d* = 0.8) (Team I & Digital N, 2020).

Functional impairment measured on the WSAS was reduced in all domains between pre‐treatment and post‐treatment, with comparable effect sizes to the publicly available reference data from IAPT (all diagnoses combined) (Team I & Digital N, 2020). In the work domain, impairment decreased from 3.04 at pre‐treatment to 2.19 at post‐treatment (*d* = 0.72 [0.54 to 0.91]), compared to a reduction from 4.2 to 2.7 (*d* = 0.6) in IAPT reference data. Home management impairment decreased from 3.35 to 2.77 (*d* = 0.52 [0.33 to 0.7]), and the reduction in the reference IAPT database was 3.5 to 2.5 (*d* = 0.4). Impairment in social leisure activities decreased from 3.65 to 2.84 (*d* = 0.71 [0.53 to 0.9]), compared to a reduction from 4.3 to 2.9 (*d* = 0.6) in IAPT reference data. Private leisure activities impairment decreased from 3.17 to 2.39 (*d* = 0.68 [0.5 to 0.87]), whereas the reduction in IAPT reference data was from 3.5 to 2.4 (*d* = 0.5). Finally, impairment in close relationships decreased from 3.05 to 2.49 (*d* = 0.51 [0.32 to 0.69]), compared to a decrease from 3.8 to 2.6 (*d* = 0.5) in the IAPT reference data.

### Recovery, improvement, and deterioration

**FIGURE 3:**
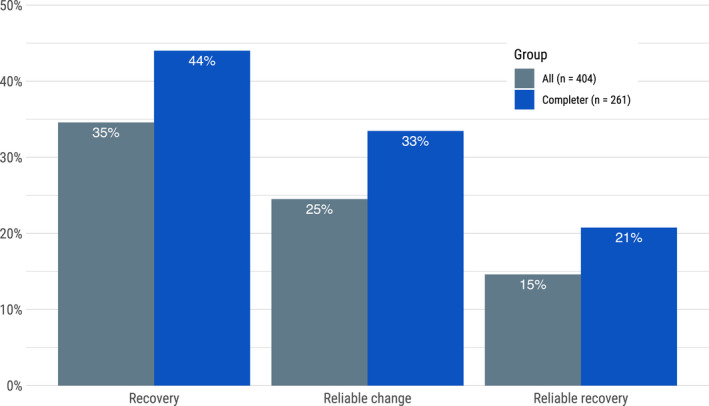
Rates of recovery, reliable change, and reliable recovery using the first and last assessments on the OCI‐R and PHQ‐9

A total of 404 participants provided at least two assessments. Among them, 138 (34.6%) were in recovery at their last assessment, that is, moving below the threshold for caseness on both the OCI‐R and PHQ‐9. The corresponding number for reliable improvement was 99 participants (24.5%), where the criteria were that either the OCI‐R or PHQ‐9 exceeds the threshold for reliable improvement while the other is also improved or unchanged. A total of 59 (14.6%) met the criteria for both recovery and improvement and were considered to be reliably recovered. Among treatment completers, the rates of recovery (44%), reliable improvement (33.5%), and reliable recovery (20.8%) were higher (Figure 3). When looking at the OCI‐R alone, 102 (25%) of participants reliably improved, 15 (4%) reliably deteriorated, and 287 (71%) neither improved nor deteriorated.

### Engagement and satisfaction

The patients sent 7.63 (*SD* = 8.91) messages on average, and received 11.24 (*SD* =8.88) messages from their therapist, however with a wide range from 0 to 50 messages sent by the participants. Out of 193 respondents on the post‐treatment patient experience questionnaire, 74% were completely or mostly satisfied with their online therapy, 80% found the online materials very helpful or helpful, 71% experienced the online therapy as easy to use, and 87% found it easy to communicate with their therapist.

## DISCUSSION

The purpose of this study was to evaluate the implementation of an evidence‐based ICBT intervention for OCD in three IAPT services across the United Kingdom. The treatment was associated with large reductions in OCD symptoms (mean reduction on the OCI‐R −10 points, *d* = 1.77), anxiety (*d* = 1.55), and depression (*d* = 0.8). Further, 35% and 25% of participants met IAPT criteria for recovery and clinically significant improvement, respectively. For outcomes where reference data from all OCD treatments in IAPT was available, OCD‐NET was associated with equal or larger improvements compared to face‐to‐face CBT which is the current gold‐standard treatment for OCD.

The results from this open trial are in line with previous evaluations of OCD‐NET, which have found effect sizes on the OCI‐R ranging from *d* = 0.9 to *d* = 1.7 (Andersson et al., 2011, 2012, 2015; Patel et al., 2018). These effect sizes are comparable or slightly higher than those obtained in other studies evaluating ICBT and low‐intensity treatments for OCD in clinical practice. For example, a large randomized controlled trial from the United Kingdom evaluated computerized CBT and guided self‐help for OCD and found pre‐treatment to post‐treatment reductions on the Yale‐Brown Obsessive–Compulsive Scale (Y‐BOCS; (Goodman et al., 1989)) of *d* = 0.6 and 0.8, respectively (observer‐rated when available otherwise self‐rated) (Lovell et al., 2017). A recent evaluation of both guided and unguided ICBT for OCD in the Australian routine care ICBT clinic *MindSpot* found standardized effect sizes on the self‐rated version of the Y‐BOCS of *g* = 0.6 at post‐treatment and *g* = 0.9 at 3‐month follow‐up (Wootton et al., 2021), similar to an early evaluation that found an effect size of *d* = 0.9 on the self‐rated version of the Y‐BOCS (Titov et al., 2017). An evaluation of a mix of guided and unguided ICBT for OCD on another online treatment platform developed in Australia, *THISWAYUP*, found a pre–post standardized effect size of *g* = 0.61 on the dimensional Y‐BOCS scale (Luu et al., 2020). Although direct comparisons between studies are hard due to differences in outcome measures used, programme structure and content, technological platforms used, and the context in which therapy is delivered, this study adds to the growing evidence base showing the effectiveness of ICBT for OCD when delivered in routine care.

This study is not without limitations. First, as this was an open trial without a control group, it is unclear whether the observed changes in symptoms were due to the treatment, general factors in care such as attention from clinicians, or due to the passage of time. However reference data of all treatments for OCD in IAPT is available as a comparison, and spontaneous remission is uncommon in OCD (Skoog & Skoog, 1999). Second, although eligibility criteria for the trial were broad to not interfere with criteria for treatment in regular clinical practice, some exclusion criteria were still in place which may reduce the generalisability of the results. For example, among the reasons for exclusion were attention deficits that would interfere with reading the treatment texts, low expressed motivation to participate, or another severe mental disorder that would interfere with treatment such as unmedicated psychosis or bipolar disorder. These, however, reflected general exclusion criteria at the IAPT services participating in the evaluation, as the services treat common mental health disorders such as depression and anxiety disorders, and not more severe conditions typically seen in specialist psychiatric care. Third, we do not have data on the number of patients who were offered OCD‐NET but declined to participate or the number of participants who were excluded prior to being registered on the online treatment platform, thus limiting the generalisability of the results to individuals who chose to participate in this evaluation over other alternatives (e.g., the waitlist for face‐to‐face treatment for OCD). Finally, OCD‐NET should be further evaluated using gold‐standard instruments such as the clinician‐administered Y‐BOCS and with longer follow‐ups, to confirm that treatment gains are maintained over time.

There are multiple challenges associated with translating findings from research into clinical practice. First, many clinicians involved in the trial had no or limited experience with OCD and were tasked with learning about effective psychological treatment for OCD and learning how to use a new treatment platform simultaneously, while also continuing their regular clinical work. To address this, regular supervision via videoconferencing was scheduled after the initial 1‐day workshop, where therapists could raise issues and questions related to the platform or the ongoing treatments. We also developed an online resource for therapists that contains written instructions and videos about using the treatment platform, as well as guidance on clinical best practices and common issues in treatment. Second, the style of communication in OCD‐NET (frequent, short messages) was unlike other digital treatments the therapists had previously encountered which were scheduled weekly or bi‐weekly, with structured sessions for 30–60 minutes at a time. During the implementation, we observed and received feedback from therapists that PWPs faced a bigger challenge than HI‐CBT in implementing the short daily check‐ins on the platform, as their schedules rarely included the 15–30 minutes blocks needed to monitor patient progress on the platform. This was also seen in post‐hoc analyses of the OCI‐R where patients treated by PWPs improved less over time. To the best of our knowledge, this is the first study to suggest that therapist seniority could be an important factor in ICBT outcome. By contrast, there was no effect of therapist experience in using OCD‐NET when comparing the first five patients to subsequent patients treated by the same therapist. HI‐CBT therapists are often expected to take on more complex patients and may in turn have more autonomy in planning their schedules, and thus be able to fit in regular checks on the OCD‐NET platform throughout the week.

## CONCLUSION

This study evaluated internet‐delivered cognitive behaviour therapy for obsessive–compulsive disorder (OCD‐NET) when implemented at three services within the IAPT programme. Improvement on self‐rated measures of OCD, depression, anxiety, and functional impairment were in line with benchmark data from all OCD treatments in IAPT. A majority of patients were satisfied with their online therapy. We conclude that OCD‐NET is an effective treatment option when implemented in IAPT services.

## CONFLICT OF INTEREST

Prof. Mataix‐Cols receives royalties for contributing articles to UpToDate, Wolters Kluwer Health, outside the submitted work. Prof. Mataix‐Cols reports personal fees from Elsevier, outside the submitted work. The other authors declare no potential conflicts of interest.

## AUTHOR CONTRIBUTION

**Oskar Flygare:** Conceptualization; Data curation; Formal analysis; Funding acquisition; Methodology; Project administration; Visualization; Writing – original draft; Writing – review & editing. **Lina Lundström:** Writing – review & editing. **Erik Andersson:** Conceptualization; Funding acquisition; Supervision; Writing – review & editing. **David Mataix‐Cols:** Conceptualization; Funding acquisition; Supervision; Writing – review & editing. **Christian Rück:** Conceptualization; Data curation; Funding acquisition; Project administration; Supervision; Writing – review & editing.

## Data Availability

The data that support the findings of this study are available on request from the corresponding author. The data are not publicly available due to privacy or ethical restrictions.

## References

[bjc12365-bib-0001] Abramowitz, J. S. , & Deacon, B. J. (2006). Psychometric properties and construct validity of the ObsessiveCompulsive InventoryRevised: Replication and extension with a clinical sample. Journal of Anxiety Disorders, 20(8), 1016–1035. 10.1016/j.janxdis.2006.03.001 16621437

[bjc12365-bib-0002] Abramowitz, J. , Tolin, D. , & Diefenbach, G. (2005). Measuring change in OCD: Sensitivity of the obsessive‐compulsive inventory‐revised. Journal of Psychopathological and Behavioral Assessment, 27(4), 317–324. 10.1007/s10862-005-2411-y

[bjc12365-bib-0003] American Psychiatric Association (2013). Diagnostic and statistical manual of mental disorders: Dsm‐5. Amer Psychiatric Pub Incorporated.

[bjc12365-bib-0004] Andersson, E. , Enander, J. , Andrén, P. , Hedman, E. , Ljótsson, B. , Hursti, T. , Bergström, J. , Kaldo, V. , Lindefors, N. , Andersson, G. , & Rück, C. (2012). Internet‐based cognitive behaviour therapy for obsessiveCompulsive disorder: A randomized controlled trial. Psychological Medicine, 42(10), 2193–2203. 10.1017/S0033291712000244 22348650 PMC3435873

[bjc12365-bib-0005] Andersson E. , Hedman E. , Enander J. , Radu Djurfeldt D. , Ljótsson B. , Cervenka S. , Isung J. , Svanborg C. , Mataix‐Cols D. , Kaldo V. , Andersson G. , Lindefors N. , & Rück C. (2015). d‐Cycloserine vs Placebo as Adjunct to Cognitive Behavioral Therapy for Obsessive‐Compulsive Disorder and Interaction With Antidepressants. JAMA Psychiatry, 72(7), 659. 10.1001/jamapsychiatry.2015.0546 25970252

[bjc12365-bib-0006] Andersson, E. , Hedman, E. , Ljótsson, B. , Wikström, M. , Elveling, E. , Lindefors, N. , Andersson, G. , Kaldo, V. , & Rück, C. (2015). Cost‐effectiveness of internet‐based cognitive behavior therapy for obsessive‐compulsive disorder: Results from a randomized controlled trial. Journal of Obsessive‐Compulsive and Related Disorders, 4, 47–53. 10.1016/j.jocrd.2014.12.004

[bjc12365-bib-0007] Andersson, E. , Ljótsson, B. , Hedman, E. , Kaldo, V. , Paxling, B. , Andersson, G. , Lindefors, N. , & Rück, C. (2011). Internet‐based cognitive behavior therapy for obsessive compulsive disorder: A pilot study. BMC Psychiatry, 11(1), 125. 10.1186/1471-244X-11-125 21812991 PMC3163522

[bjc12365-bib-0008] Andersson, E. , Steneby, S. , Karlsson, K. , Ljótsson, B. , Hedman, E. , Enander, J. , Kaldo, V. , Andersson, G. , Lindefors, N. , & Rück, C. (2014). Long‐term efficacy of Internet‐based cognitive behavior therapy for obsessiveCompulsive disorder with or without booster: A randomized controlled trial. Psychological Medicine, 44(13), 2877–2887. 10.1017/S0033291714000543 25066102

[bjc12365-bib-0009] Bennion, M. R. , Hardy, G. , Moore, R. K. , & Millings, A. (2017). E‐therapies in England for stress, anxiety or depression: What is being used in the NHS? A survey of mental health services. British Medical Journal Open, 7(1), e014844. 10.1136/bmjopen-2016-014844 PMC5278266 28115336

[bjc12365-bib-0010] Clark, D. M. (2011). Implementing NICE guidelines for the psychological treatment of depression and anxiety disorders: The IAPT experience. International Review of Psychiatry, 23(4), 318–327. 10.3109/09540261.2011.606803 22026487 PMC3212920

[bjc12365-bib-0011] Clark, D. M. (2018). Realizing the mass public benefit of evidence‐based psychological therapies: The IAPT program. Annual Review of Clinical Psychology, 14(1), 159–183. 10.1146/annurev-clinpsy-050817-084833 PMC5942544 29350997

[bjc12365-bib-0012] Clark, D. M. , Canvin, L. , Green, J. , Layard, R. , Pilling, S. , & Janecka, M. (2018). Transparency about the outcomes of mental health services (IAPT approach): An analysis of public data. The Lancet, 391(10121), 679–686. 10.1016/S0140-6736(17)32133-5 PMC5820411 29224931

[bjc12365-bib-0013] England, P. H. (2021). Common mental health disorders ‐ PHE. Retrieved from: https://fingertips.phe.org.uk/profile‐group/mental‐health/profile/common‐mental‐disorders

[bjc12365-bib-0014] Fawcett, E. J. , Power, H. , & Fawcett, J. M. (2020). Women are at greater risk of OCD than men: A meta‐analytic review of OCD prevalence worldwide. The Journal of Clinical Psychiatry, 81(4). 10.4088/JCP.19r13085 32603559

[bjc12365-bib-0015] Foa, E. B. , Huppert, J. D. , Leiberg, S. , Langner, R. , Kichic, R. , Hajcak, G. & Salkovskis, P. M. (2002). The obsessive‐compulsive inventory: Development and validation of a short version. Psychological Assessment, 14(4), 485–496. 10.1037/1040-3590.14.4.485 12501574

[bjc12365-bib-0016] Foa, E. B. , Yadin, E. , & Lichner, T. K. (2012). Exposure and response (ritual) prevention for obsessive‐compulsive disorder: Therapist guide (2nd ed.). Oxford University Press. (Treatments that work).

[bjc12365-bib-0017] Fullana, M. A. , Mataix‐Cols, D. , Caspi, A. , Harrington, H. , Grisham, J. R. , Moffitt, T. E. & Poulton, R. (2009). Obsessions and compulsions in the community: prevalence, interference, help‐seeking, developmental stability, and co‐occurring psychiatric conditions. American Journal of Psychiatry, 166(3), 329–336. 10.1176/appi.ajp.2008.08071006 19188283 PMC3818089

[bjc12365-bib-0018] Goodman, W. K. , Price, L. H. , Rasmussen, S. A. , Mazure, C. , Delgado, P. , Heninger, G. R. , & Charney, D.S. (1989). The Yale‐Brown Obsessive Compulsive Scale. I. Development, Use, and Reliability. Archives of General Psychiatry, 46(11), 1012–1016. 10.1001/archpsyc.1989.01810110054008 2510699

[bjc12365-bib-0019] Goodwin, R. , Koenen, K. C. , Hellman, F. , Guardino, M. , & Struening, E. (2002). Helpseeking and access to mental health treatment for obsessive‐compulsive disorder. Acta Psychiatrica Scandinavica, 106(2), 143–149. 10.1034/j.1600-0447.2002.01221.x 12121213

[bjc12365-bib-0020] Hedman, E. , Ljótsson, B. , & Lindefors, N. (2012). Cognitive behavior therapy via the Internet: A systematic review of applications, clinical efficacy and costEffectiveness. Expert Review of Pharmacoeconomics & Outcomes Research, 12(6), 745–764. 10.1586/erp.12.67 23252357

[bjc12365-bib-0021] Hoppen, L. M. , Kuck, N. , Bürkner, P.‐C. , Karin, E. , Wootton, B. M. , & Buhlmann, U. (2021). Low intensity technology‐delivered cognitive behavioral therapy for obsessive‐compulsive disorder: A meta‐analysis. BMC Psychiatry, 21(1), 322. 10.1186/s12888-021-03272-5 34193113 PMC8243493

[bjc12365-bib-0022] Huppert, J. D. , Simpson, H. B. , Nissenson, K. J. , Liebowitz, M. R. , & Foa, E. B. (2009). Quality of life and functional impairment in obsessiveCompulsive disorder: A comparison of patients with and without comorbidity, patients in remission, and healthy controls. Depression and Anxiety, 26(1), 39–45. 10.1002/da.20506 18800368 PMC2707595

[bjc12365-bib-0023] Kroenke, K. , Spitzer, R. L. , & Williams, J. B. (2001). The PHQ‐9: Validity of a brief depression severity measure. Journal of General Internal Medicine, 16(9), 606–613. 10.1046/j.1525-1497.2001.016009606.x 11556941 PMC1495268

[bjc12365-bib-0024] Lovell, K. , Bower, P. , Gellatly, J. , Byford, S. , Bee, P. , McMillan, D. , Arundel, C. , Gilbody, S. , Gega, L. , Hardy, G. , Reynolds, S. , Barkham, M. , Mottram, P. , Lidbetter, N. , Pedley, R. , Molle, J. , Peckham, E. , Knopp‐Hoffer, J. , Price, O. , Connell, J. , Heslin, M. , Foley, C. , Plummer, F. , & Roberts, C. (2017). Low‐intensity cognitive‐behaviour therapy interventions for obsessive‐compulsive disorder compared to waiting list for therapist‐led cognitive‐behaviour therapy: 3‐arm randomised controlled trial of clinical effectiveness. PLoS Med, 14(6), e1002337. 10.1371/journal.pmed.1002337 28654682 PMC5486961

[bjc12365-bib-0025] Luu, J. , Millard, M. , Newby, J. , Haskelberg, H. , Hobbs, M. J. , & Mahoney, A. E. J. (2020). Internet‐based cognitive behavioural therapy for treating symptoms of obsessive compulsive disorder in routine care. Journal of Obsessive‐Compulsive and Related Disorders, 26, 100561. 10.1016/j.jocrd.2020.100561

[bjc12365-bib-0026] Marques, L. , LeBlanc, N. J. , Weingarden, H. M. , Timpano, K. R. , Jenike, M. , & Wilhelm, S. (2010). Barriers to treatment and service utilization in an internet sample of individuals with obsessiveCompulsive symptoms. Depression and Anxiety, 27(5), 470–475. 10.1002/da.20694 20455248

[bjc12365-bib-0027] Mataix‐Cols, D. , Rauch, S. L. , Baer, L. , Eisen, J. L. , Shera, D. M. , Goodman, W. K. , Rasmussen, S. A. , & Jenike, M. A. (2002). Symptom stability in adult obsessive‐compulsive disorder: data from a naturalistic two‐year follow‐up study. American Journal of Psychiatry, 159(2), 263–268. 10.1176/appi.ajp.159.2.263 11823269

[bjc12365-bib-0028] Mundt, J. C. , Marks, I. M. , Shear, M. K. , & Greist, J. M. (2002). The Work and Social Adjustment Scale: A simple measure of impairment in functioning. British Journal of Psychiatry, 180(5), 461–464. 10.1192/bjp.180.5.461 11983645

[bjc12365-bib-0029] NICE (2019). OCD‐NET for adults with obsessive compulsive disorder. National Institute for Health and Care Excellence. 31869034

[bjc12365-bib-0030] National Institute for Clinical Excellence (2005). Obsessive‐Compulsive disorder: Core interventions in the treatment of obsessive‐compulsive disorder and body dysmorphic disorder. OCD. Retrieved from: https://www.nice.org.uk/guidance/cg31 31886982

[bjc12365-bib-0031] Öst, L.‐G. , Havnen, A. , Hansen, B. , & Kvale, G. (2015). Cognitive behavioral treatments of obsessiveCompulsive disorder. A systematic review and meta‐analysis of studies published 1993. Clinical Psychology Review, 40, 156–169. 26117062 10.1016/j.cpr.2015.06.003

[bjc12365-bib-0032] Patel, S. R. , Wheaton, M. G. , Andersson, E. , Rück, C. , Schmidt, A. B. , La Lima, C. N. , Galfavy, Hanga , Pascucci, O. , Myers, R. W. , Dixon, L. B. , & Simpson, H. B. (2018). Acceptability, feasibility, and effectiveness of internet‐based cognitive‐behavioral therapy for obsessive‐compulsive disorder in New York. Behavior Therapy, 49(4), 631–641. 10.1016/j.beth.2017.09.003 29937263 PMC6945297

[bjc12365-bib-0033] Pérez‐Vigil, A. , Fernández de la Cruz, L. , Brander, G. , Isomura, K. , Jangmo, A. , Feldman, I. , Hesselmark, E. , Serlachius, E. , Lázaro, L. , Rück, C. , Kuja‐Halkola, R. , D’Onofrio, B. M. , Larsson, H. , & Mataix‐Cols, D. (2018). Association of obsessive‐compulsive disorder with objective indicators of educational attainment: A nationwide register‐based sibling control study. JAMA Psychiatry, 75(1), 47. 10.1001/jamapsychiatry.2017.3523 29141084 PMC5833536

[bjc12365-bib-0034] Pérez‐Vigil, A. , Mittendorfer‐Rutz, E. , Helgesson, M. , de la Cruz, L. F. , & Mataix‐Cols, D. (2018). Labour market marginalisation in obsessiveCompulsive disorder: A nationwide register‐based sibling control study. Psychological Medicine, 49(6), 1015–1024. 10.1017/S0033291718001691 29950186

[bjc12365-bib-0035] R Core Team. R (2021). A language and environment for statistical computing. R Foundation for Statistical Computing.

[bjc12365-bib-0036] Richards, D. , Duffy, D. , Blackburn, B. , Earley, C. , Enrique, A. , Palacios, J. et al. (2018). Digital IAPT: The effectiveness & cost‐effectiveness of internet‐delivered interventions for depression and anxiety disorders in the Improving Access to Psychological Therapies programme: Study protocol for a randomised control trial. BMC Psychiatry, 18(1), 59. 29499675 10.1186/s12888-018-1639-5 PMC5833053

[bjc12365-bib-0037] Rück, C. , Lundström, L. , Flygare, O. , Enander, J. , Bottai, M. , Mataix‐Cols, D. & Andersson, E. (2018). Study protocol for a single‐blind, randomised controlled, non‐inferiority trial of internet‐based versus face‐to‐face cognitive behaviour therapy for obsessiveCompulsive disorder. British Medical Journal Open, 8(9), e022254. 10.1136/bmjopen-2018-022254 PMC6129083 30185575

[bjc12365-bib-0038] Schröder, J. , Werkle, N. , Cludius, B. , Jelinek, L. , Moritz, S. , & Westermann, S. (2020). Unguided Internet‐based cognitive‐behavioral therapy for obsessive‐compulsive disorder: A randomized controlled trial. Depression and Anxiety, 37(12), 1208–1220. 10.1002/da.23105 33169490

[bjc12365-bib-0039] Skoog, G. , & Skoog, I. (1999). A 40‐year follow‐up of patients with obsessive‐compulsive disorder. Archives of General Psychiatry, 56(2), 121. 10.1001/archpsyc.56.2.121 10025435

[bjc12365-bib-0040] Spitzer, R. L. , Kroenke, K. , Williams, J. B. W. , & Löwe, B. (2006). A brief measure for assessing generalized anxiety disorder: The GAD‐7. Archives of Internal Medicine, 166(10), 1092. 10.1001/archinte.166.10.1092 16717171

[bjc12365-bib-0041] Team I, Digital N (2020). Psychological therapies, annual report on the use of IAPT services 2019‐20. NHS Digital.

[bjc12365-bib-0042] The National Collaborating Centre for Mental Health (2021). The improving access to psychological therapies manual. The National Collaborating Centre for Mental Health. Aug. Report No.: 5.

[bjc12365-bib-0043] Thew, G. R. (2020). IAPT and the internet: the current and future role of therapist‐guided internet interventions within routine care settings. The Cognitive Behaviour Therapist, 13, E4. 10.1017/S1754470X20000033 34567240 PMC8442601

[bjc12365-bib-0044] Titov, N. , Dear, B. F. , Staples, L. G. , Bennett‐Levy, J. , Klein, B. , Rapee, R. M. , Andersson, G. , Purtell, C. , Bezuidenhout, G. , & Nielssen, O. B. (2017). The first 30 months of the MindSpot Clinic: Evaluation of a national e‐mental health service against project objectives. Australian & New Zealand Journal of Psychiatry, 51(12), 1227–1239. 10.1177/0004867416671598 27733709

[bjc12365-bib-0045] Veale, D. , Lim, L. F. , Nathan, S. L. , & Gledhill, L. J. (2016). Sensitivity to change in the Obsessive Compulsive Inventory: Comparing the standard and revised versions in two cohorts of different severity. Journal of Obsessive‐Compulsive and Related Disorders, 9, 16–23. 10.1016/j.jocrd.2016.02.001

[bjc12365-bib-0046] Vogel, P. A. , Solem, S. , Hagen, K. , Moen, E. M. , Launes, G. , Håland, Å. T. , Hansen, B. , & Himle, J. A. (2014). A pilot randomized controlled trial of videoconference‐assisted treatment for obsessive‐compulsive disorder. Behaviour Research and Therapy, 63, 162–168. 10.1016/j.brat.2014.10.007 25461792

[bjc12365-bib-0047] Wootton, B. M. , Johnston, L. , Dear, B. F. , Terides, M. D. , & Titov, N. (2013). Remote treatment of obsessive‐compulsive disorder: A randomized controlled trial. J Obsessive Compuls Relat Disord, 2(4), 375–384. 10.1016/j.jocrd.2013.07.002

[bjc12365-bib-0048] Wootton, B. M. , Karin, E. , Dear, B. F. , Staples, L. , Nielssen, O. , Kayrouz, R. & Titov, N. (2021). Internet‐delivered cognitive‐behaviour therapy (ICBT) for obsessive‐compulsive disorder when delivered as routine clinical care: A phase IV clinical trial. Journal of Anxiety Disorders, 82, 102444. 10.1016/j.janxdis.2021.102444 34273871

